# A forum for global population health, technological advances and implementation science

**DOI:** 10.1017/gheg.2015.2

**Published:** 2016-02-05

**Authors:** M. S. Sandhu

**Affiliations:** 1 Department of Medicine, University of Cambridge, Cambridge, UK; 2 Sandhu l Global Health and Populations Group, Human Genetics, Wellcome Trust Sanger Institute, Hinxton, UK

Welcome to *Global Health*, Epidemiology *& Genomics* (*GHEG*), a new Open Access journal committed to providing a forum for communicating research and views on interdisciplinary global health.

GHEG welcomes research contributions that address and increase our understanding of human population diversity, health and disease, with a special emphasis on those that describe advances in technologies [1] and implementation science [2] relevant to global health and disadvantaged populations. In the face of emerging epidemics, neglected diseases, drug resistance and complex epidemiological transitions, we need to improve access to and delivery of healthcare on a global scale, including integrated healthcare systems for both communicable and non-communicable diseases and co-morbidities at a cost that can be borne by nations with limited resourcing and within the most challenging environments [3]. To facilitate the integration of research findings and evidence into healthcare policy and practice, we will also need to assimilate discovery science and technological advances, including new drugs, vaccines, and methods for disease surveillance and diagnosis. Such advances have the potential to save millions of lives, globally.

In these contexts, GHEG will disseminate a broad range of research and views on global health. We aim to promote research that spans genetic disease susceptibility and genomic medicine [4], pathogen surveillance and disease transmission, to the integration of e-health resources, mobile technologies, point-of-care testing for diseases [1], healthcare systems and delivery, and pragmatic community or therapeutic interventions to improve global human health. The breadth of this journal will be its strength.

As an Open Access and on-line journal, we encourage readers to access articles outside the immediate scope of their own work and thereby increase and strengthen knowledge beyond their individual specialties. We believe that by building an interactive, open and welcoming community of global health research and implementation professionals we can increase the level of interest, understanding and commitment to work in the global health arena. To that end, alongside the journal, we have developed an active website featuring interviews, blog articles and social media comments. We encourage our readership to actively contribute in this area.

For ease of navigation, we have categorised contributions to GHEG by subject matter covering a comprehensive range of relevant areas including epidemiology, genetics, observational epidemiological studies, community interventions, clinical trials, health systems and services, and population demography and history. Given that many articles will have multidisciplinary themes, we will link articles across research themes, disciplines and by geographic region. These future developments will help identify knowledge gaps and research needs. We also plan to publish themed collections of the journal to allow for detailed exploration of topical and current areas relevant to the global health agenda. These will be announced through a call for papers and will be led by guest editors. We are already working on two themed editions that will focus on the health of Indigenous populations and work being undertaken by the Human Heredity and Health in Africa (H3Africa) Consortium (http://www.h3africa.org [5]).

To complement the breadth of GHEG's scope its editorial team has purposefully been selected to represent the full scope of its interests. The Associate Editors offer an impressive wealth of experience and expertise—spanning public health, epidemiology, genomics, Indigenous health, infectious and non-communicable disease in a global context—and reflect the worldwide focus of GHEG, representing Africa, Asia, Australasia, Europe, North America and South America.

GHEG is a journal for the worldwide health community and is launched as an Open Access publication to ensure its content is accessible and freely available. It aspires to be truly global in content and seeks and encourages submissions from all nations. As evidence of our commitment to this aim, the journal actively supports the provision of language support services and will waive all article processing charges until December 2016. From 2017, we will offer a waiver system for eligible countries. All articles remain the property of the authors, and reuse by the wider research community and beyond will fall under a range of creative common licences.

I hope you will agree that the launch of GHEG is an exciting and timely venture. However, if it is to fulfil its promise, to become a valuable, practical and informative resource, it requires active engagement by its readership. We therefore encourage you to participate in and contribute towards the discussion about global health, genomics and epidemiology and look forward to hearing your thoughts on the journal.
